# Pulsed Irradiation Improves Target Selectivity of Infrared Laser-Evoked Gene Operator for Single-Cell Gene Induction in the Nematode *C. elegans*

**DOI:** 10.1371/journal.pone.0085783

**Published:** 2014-01-20

**Authors:** Motoshi Suzuki, Naoya Toyoda, Shin Takagi

**Affiliations:** Division of Biological Science, Graduate School of Science, Nagoya University, Nagoya, Japan; Inserm U869, France

## Abstract

Methods for turning on/off gene expression at the experimenter’s discretion would be useful for various biological studies. Recently, we reported on a novel microscope system utilizing an infrared laser-evoked gene operator (IR-LEGO) designed for inducing heat shock response efficiently in targeted single cells in living organisms without cell damage, thereby driving expression of a transgene under the control of a heat shock promoter. Although the original IR-LEGO can be successfully used for gene induction, several limitations hinder its wider application. Here, using the nematode *Caenorhabditis elegans* (*C. elegans*) as a subject, we have made improvements in IR-LEGO. For better spatial control of heating, a pulsed irradiation method using an optical chopper was introduced. As a result, single cells of *C. elegans* embryos as early as the 2-cell stage and single neurons in ganglia can be induced to express genes selectively. In addition, the introduction of site-specific recombination systems to IR-LEGO enables the induction of gene expression controlled by constitutive and cell type-specific promoters. The strategies adopted here will be useful for future applications of IR-LEGO to other organisms.

## Introduction

Ectopic transgene expression is a powerful means for analyzing gene functions as well as for manipulating cells in multicellular organisms *in vivo*. The use of tissue-specific promoters for ectopic expression, however, is restricted by their inherent properties to drive gene expression in a limited spatiotemporal expression pattern. One strategy for the temporal control of transgene expression exploits the conserved heat shock response, in which heat stress induces the expression of heat shock proteins [1], [2]. A transgene under the control of a heat shock promoter can be induced following a temperature shift at the desired time. To lend spatial resolution to the heat shock response, local heating has been attempted by irradiation with microbeams of visible laser light using cell-ablation microscope systems [3], [4], [5], [6]. This method, however, has not been commonly used, because irradiation often damages cells and induces genes inefficiently. For the efficient and harmless heating of single targeted cells, a novel microscope system utilizing an infrared laser-evoked gene operator (IR-LEGO) was recently developed [7]. The application of IR-LEGO on a *Caenorhabditis elegans* (*C. elegans*) nematode and other organisms proved that the expression of genes can be induced efficiently in a finely regulated temporal-spatial manner in living organisms with minimal detrimental effects [7], [8], [9].

The model animal *C. elegans* is suited for laser-mediated gene induction experiments. Its small transparent body works well for observation and operation under a microscope. The complete cell lineage of the 959 adult somatic cells was determined and found to be remarkably consistent among animals [10], [11]. The anatomy of the nervous system was also described through serial electron microscopic reconstruction at the synaptic level [12], leading to thorough documentation of the connectivity among all identifiable 302 neurons. These features not only permit the unambiguous identification of target cells for laser irradiation, but also give us an unparalleled opportunity to clarify how genes dictate the form and behavior of this organism at single-cell resolution. Therefore, the manipulation of gene expression at the single cell level offers a unique advantage in *C. elegans* studies. Previous attempts to manipulate gene expression in single *C. elegans* neurons had limited success because few cell-specific promoters have been identified for neurons. Only 12% of the 118 neuronal groups studied by White et al. [12] can be forced to express transgenes selectively using the available individual cell-specific promoters [13]. Combinatorial use of promoters would help to achieve higher cell-type selectivity, even to single neuronal groups [14], yet genetic manipulation of an individual neuron within a neuronal group cannot be attained. Given the recent development of tools for modulating neuronal activity, such as light-activated channels/pumps [15], [16] and tetanus toxin [17], the application of IR-LEGO in neurobiological studies seems even more promising.

The current IR-LEGO technology has several limitations. First, not all *C. elegans* cells can be induced selectively. We previously showed successful gene induction with continuous IR irradiation in targeted single cells of several cell types, including epidermal cells, body wall muscle cells, lateral body neurons, and migratory distal tip cells (DTCs) of the gonad, which are located either on or close to the body wall of worms [7]. On the other hand, cells located deep within cell clusters, such as neurons in ganglia, are difficult targets. Multiple cells in the vicinity of the target were usually also induced, indicating insufficiency in the spatial control of heating. Furthermore, gene expression in early embryonic cells cannot be induced efficiently. Thus, improvements in the irradiation method to enable more spatially localized and finely tuned heating are needed. Second, heat shock-induced gene expression occurs only transiently, though sustained gene expression is more advantageous for experiments such as cell lineage analyses. Third, heat shock promoters generally drive robust gene expression. The expression level of the induced gene cannot be controlled, and overexpression may obscure its physiologically pertinent function. Thus, a means for controlling the expression in more elaborate manners, e.g., those mimicking the expression pattern of the endogenous gene, is required. To overcome these problems, we have introduced technical modifications, a pulsed irradiation protocol, and exogenous site-specific recombination systems to the original IR-LEGO.

## Results and Discussion

### Improved Spatial Control of Gene Induction with Pulsed Irradiation

**Figure 1:**
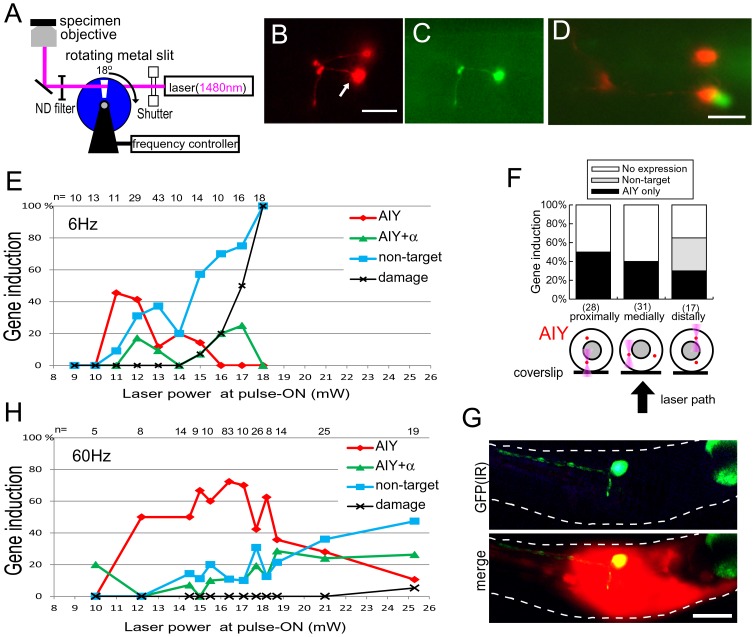
Gene induction in targeted single neurons by pulsed IR irradiation. (A) Schematic of IR-LEGO for pulsed irradiation with an external chopper in the laser path. (B) A pair of AIY neurons (AIYR and AIYL) in an adult *ncIs201* worm visualized with *ttx-3p::mRFP*. (C) AIYR in *ncIs201* (arrow in B) was irradiated with 833 µsec laser pulses for 4 s at 60 Hz at an incident power of 11 mW. GFP expression was detected in the target 3 h later. (D) GFP expression was sometimes detected in a neuron near the target. Note that GFP is induced in only a single neuron. (E) Gene induction index against incident laser power during the pulse-on period, for irradiation with 8.3 ms laser pulses for 4 s at 6 Hz on a single AIY in *ncIs201*. In this experiment, a single AIY was irradiated randomly, irrespective of its position. Red diamonds indicate GFP expression in only the target cell; green triangles indicate GFP expression in both the target single cell and a non-target cell. Blue squares indicate the gene expression index in only a non-target cell. Gene induction was 3 h after irradiation. Numbers of trials are shown at the upper margin. (F) Schematic showing the position of targeted AIY, which are located proximally, medially, and distally from the objective from left to right (bottom). Histograms show gene induction patterns in AIY for three different positions of pulsed irradiation at 12 mW. Black, gray, and white bars represent GFP expression in AIY alone, GFP expression in another cell alone, and no GFP expression, respectively (top). (G) A single neuron in *ncIs17* was IR-irradiated under the same conditions. GFP expression was detected in its soma and in a long anterior and a short ventral process. Neurons in the nerve ring are also labeled with a pan-neuronal marker transgene *H20::mrfp.* (H) Gene induction index against incident laser power during the pulse-on period, for irradiation with 833 µs laser pulses for 4 s at 60 Hz on a single AIY in *ncIs201*. Induction was scored and represented similarly to (E). (B, D, and G) Scale bars = 10 µm.

We showed previously that irradiation with IR-LEGO successfully induced gene expression in a single ALM touch neuron on the body wall, proving that IR-LEGO can be applied to the nervous system [7]. When we irradiated a single neuron in the nerve ring where many neurons and their processes are packed closely together, however, gene induction often occurred in multiple cells (see the next section), suggesting that the continuous irradiation employed in IR-LEGO raised the temperature of the surroundings of the target above the threshold of heat shock induction. To improve the spatial control of heating, we installed an external chopper in the laser path in order to irradiate cells with brief pulses instead of continuous irradiation (Fig. 1A). As discussed by Bargmann and Avery [18], heat diffusing away from the laser focus will reach a steady state with continuous irradiation, whereas with irradiation delivered in brief pulses, the steady state will not be achieved and heat will not accumulate in the area surrounding the focus, given sufficient time for heat dissipation between pulses. A similar strategy was adopted for laser-mediated gene induction in butterflies [19].

The IR laser beam was modified to irradiate cells with 8.3 ms pulses at a frequency of 6 Hz. First, we compared the effects of continuous and pulsed irradiation on epidermal cells. Seam cells in *ncIs17[hsp16-2::gfp]* worms, which carry GFP cDNA under the control of a heat shock promoter, at the fourth larval stage (L4) were irradiated with the laser at various power levels. With continuous irradiation for 4 s, gene induction in target cells (Fig. S1A) sometimes accompanied gene induction in non-target cells, mostly body wall muscle cells beneath the target, as shown previously (Fig. S1B, C). On the other hand, with pulsed irradiation for 4 s, gene induction was restricted nearly exclusively to targeted cells (Fig. S1D). With an incident laser power level of 20 mW during a pulse-on period, the irradiated seam cell was induced to express GFP exclusively in 50% of cases. At higher power, irradiated cells sometimes appeared to be damaged, demonstrating that irradiation, even in pulses, at an excessive power level is harmful to cells, as expected. Notably, even under such conditions, GFP expression was rarely detected in non-targeted cells, indicating that heat is indeed confined to the target more locally with pulsed irradiation, leading to higher target selectivity.

### Gene Induction in Single Neurons in Cell Clusters with Pulsed Irradiation

We tested whether pulsed IR irradiation is suitable for single-cell gene induction in cell clusters. AIYs (AIYL and AIYR), a pair of interneurons located bilaterally in the nerve ring, were visualized with *mRFP* in *ncIs201[hsp16-2::gfp, ttx-3p::mrfp]* for irradiation (Fig. 1B). First, we targeted AIYs randomly, irrespective of their relative position to the objective. With continuous irradiation, despite extensive attempts under varying conditions, we failed to induce gene exclusively in AIY. Expression of GFP was mainly observed in body wall muscles, pharyngeal muscles, and non-targeted neurons in the nerve ring (data not shown). Next, we performed irradiation with 8.3 ms laser pulses for 4 s at 6 Hz at various power levels. With an incident laser power of 11 mW during a pulse-on period, approximately 40% of irradiated AIYs expressed GFP (Fig. 1E). Induction occurred almost exclusively in AIY (Fig. 1C), but in some cases GFP was expressed in a non-targeted single neuron close to AIY (Fig. 1D). At an incident laser power higher than 15 mW, cell damage became detectable (Fig. 1E); irradiated neurons sometimes changed their shape and mRFP fluorescence vanished 3 h after irradiation. When AIY was damaged, GFP expression was often observed in neighboring cells but not in AIY.

Next, we examined the relationships between the target-selectivity of gene induction and the depth of AIY in specimens. When worms were mounted with their ventral side facing the objective in order to position AIY medially in the body, approximately 40% of AIY irradiated at 12 mW expressed GFP exclusively (Fig. 1F). When worms were mounted with their lateral side facing the objective and the AIY located proximally to the objective was targeted, approximately 50% of the irradiated AIY expressed GFP with expression in no other cells (Fig. 1F). When the distally located AIY was targeted, GFP was expressed in the targeted AIY in 30% of cases. However, GFP was also expressed in a nearby non-irradiated cell in 35% of cases without GFP expression in AIY (Fig. 1F). Thus, the nearer the target is to the objective, the higher the target-selectivity of gene induction. Even when irradiation of distal AIY missed the target, it is notable that gene induction occurred almost exclusively in a single cell close to the target, indicating that heat deposition is sufficiently localized for single-cell gene induction at a depth of approximately 50 µm. Under the same conditions, we also succeeded in inducing genes in other single neurons in the nerve ring (Fig. 1G).

We also tested the pulsed IR irradiation with a shorter pulse-on period at a higher frequency: 833 µs pulses at 60 Hz for 4 s. As shown in Fig. 1H, induction that was highly selective to targeted AIY occurred in a wider range of power levels compared to irradiation with 8.3 ms pulses at 6 Hz. Targeted cells expressed GFP in 70% of cases at 17.1 mW. Cell damage was not observed at power levels below 25.3 mW, but all irradiated cells were damaged at 50.6 mW (n = 5). When AVA, another interneuron in the nerve ring, was targeted under the same pulse condition during a pulse-on period with the power level at 17.1 mW, we observed induction exclusive to the targeted neuron in 40% of cases (data not shown). Although we have not individually tested all 302 neurons of *C. elegans*, it seems plausible that most of them are amenable to gene induction with modified LEGO.

### Gene Induction in Targeted Single Cells of Early Embryos

**Figure 2:**
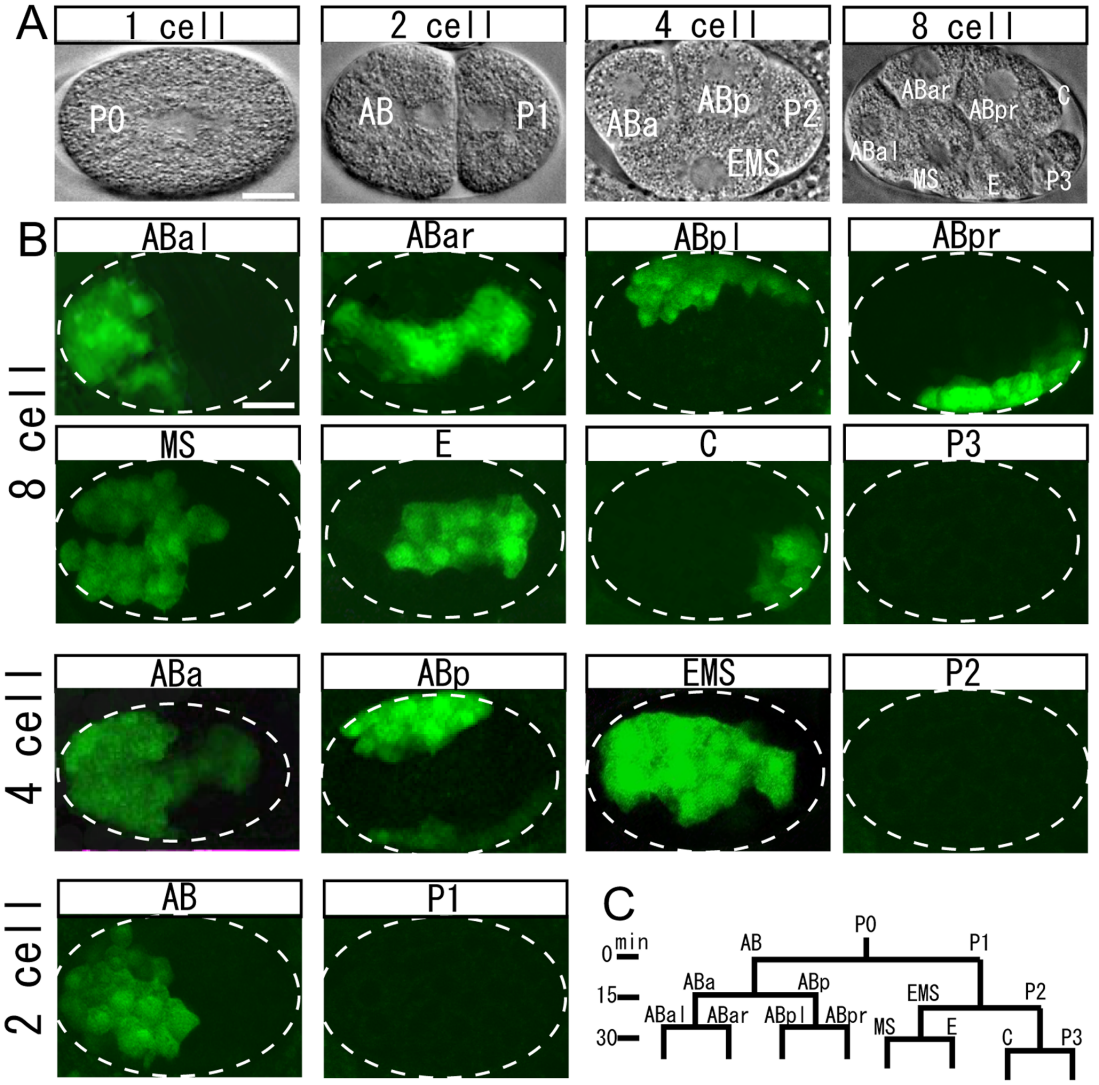
Gene induction in targeted single blastomeres. (A) *C. elegans* early embryos viewed with Nomarski optics. (B) GFP expression 3 h after irradiation of single blastomeres of 8-, 4-, and 2-cell embryos. Irradiation of ABar, ABal, ABpr, ABpl, MS, E, and C in 8-cell embryos, and of ABa, ABp, and EMS in 4-cell embryos lead to GFP expression in a cell cluster corresponding to the descendants of the respective blastomere in embryos in the late blastula stage. Irradiation of AB in 2-cell embryos leads to variable GFP expression patterns in AB descendants. Irradiation of germ-line blastomeres P1 and P2 mostly failed to induce GFP induction. Scale bar = 10 µm. (C) Lineage pattern of early cleavage in the *C. elegans* embryo.

In previous attempts to target early embryonic cells using continuous IR irradiation, gene induction occurred only infrequently and irradiated cells often failed to develop. We tested pulsed IR irradiation, which permits heating in a more elaborate manner, on embryos, and found that single cells in early embryos were successfully induced (Fig. 2, S2). The optimum condition for gene induction in embryos differed significantly depending on their stages, as described below. In general, the laser power required for gene induction was positively correlated with the size of the cells. For some blastomeres, application of pulsed irradiation multiple times is effective. Embryos irradiated under appropriate conditions developed normally.

We first irradiated single cells at random in *ncIs17[hsp16-2::gfp]* embryos from the 2-cell to 16-cell stage for 4 s with 8.3 ms laser pulses at 6 Hz at various power levels (Fig. S2A). With irradiation of an early blastomere, successful gene induction was evidenced by GFP expression in a cluster of its descendants at the late gastrula or comma stage. The induction marker GFP becomes detectable 3 h after irradiation while early embryos undergo cell division around every 15 min (Fig. 2C). IR irradiation at powers below 15 mW during a pulse-on period failed to induce GFP expression in early embryos. By irradiation of embryos at the 8- and 16-cell stages at 16 mW, GFP expression was sometimes detected in the descendants of single blastomeres. At 17 mW, approximately 50% of blastomeres in 16-cell embryos were induced to express GFP. At 18 mW, 66% of blastomeres in 8-cell embryos were induced to express GFP. At 21 mW, 30% of blastomeres in 4-cell embryos were induced to express GFP. Under these conditions, all irradiated embryos reached adulthood. At power levels higher than 19 mW, 20 mW, and 22 mW, some embryos at the 16-, 8-, and 4-cell stages, respectively, failed to reach adulthood following irradiation, indicating that the irradiated cells were damaged and the development of embryos was hindered. In some arrested embryos, irradiated cells assumed aberrant morphology and stopped cell division immediately. In most of the arrested embryos, GFP induction was detected. In 2-cell embryos, expression of GFP was induced only rarely.

When single cells in comma-stage embryos were targeted randomly, we observed that single cells in embryos were sometimes induced to express GFP (Fig. S2C, Table S1). It is, however, unclear whether the targeted cells in comma-stage embryos were indeed induced, as we neither identified the target cells at irradiation, nor the GFP-expressing cells in the later embryos. We found that *ncIs17* embryos were mostly at the 1.5- to 2-fold stage 3 h after irradiation, indicating that, compared to wild type animals, development of these embryos was retarded after irradiation for unknown reasons, though many of them eventually reached maturity.

Next, we targeted each individual blastomere of the embryos. In 8-cell embryos, blastomeres producing exclusively somatic lineage cells, i.e., ABar, ABal, ABpr, ABpl, MS, E, and C, were consistently (55%, n = 102) induced with the power level at 18 mW during a pulse-on period, whereas the germ-line blastomere, P3, was not (0%, n = 23) (Fig. 2B). For 2- and 4-cell embryos, we found that gene expression can be induced more efficiently by applying pulsed irradiation for 4 s twice with a 1 s interval between applications. In 4-cell embryos, ABa, ABp, and EMS somatic blastomeres were induced at 18 mW in 40% of cases (n = 15) (Fig. 2B). When the germ-line blastomere P2 was irradiated before the division of its companion ABa and ABp blastomeres, GFP expression was not induced (Fig. 2B, C). However, by irradiation of P2 after the division of ABa and ABp, we found GFP expression in a cluster of cells corresponding to C descendants (37%, n = 19) (Fig. S2B). In 2-cell embryos, irradiation at 23 mW of the somatic blastomere AB led to GFP expression in 30% of cases (n = 20) in variable patterns in later stages. Some embryos expressed GFP in all AB descendants, whereas others expressed GFP in combinations of the descendants of ABal, ABar, ABp, or ABp (Fig. 2B, S2B). Irradiation of the germ-line blastomere P1 prior to the division of its companion AB blastomere resulted in no GFP expression, but irradiation after the division of AB led to GFP expression in EMS descendants (Fig. 2B, S2B).

Previous studies using whole-body heat shock treatment suggested that the heat shock promoter can be induced in *C. elegans* embryos as early as the 12-cell stage [20]. Showing the heat shock-mediated gene induction in earlier embryos, our findings proved that IR-LEGO would be useful for studying early embryogenesis. Unsuccessful induction of GFP expression by irradiation of P3 is consistent with the known germ-line silencing of transgenes in repetitive arrays [21]. Worms with integrated single-copy transgenes would be useful for gene induction in single germ-line cells. Unexpectedly, we occasionally found GFP expression in C descendants following irradiation of the germ-line blastomere P2, which suggests the following scenario: Usually, heat shock factors activated by IR irradiation immediately enter the nucleus of irradiated cells where they promote transcription of their target genes. When activated by irradiation immediately prior to the cleavage of P2, heat shock factors would be distributed to both of its daughter cells. In C, this leads to an induction of transcription of the target genes, whereas in P3, transcription is silenced. Likewise, irradiation of P1 immediately prior to its division would lead to the distribution of activated heat shock factors to EMS.

### Induction of Site-specific Recombination in *C. elegans*

Next, we attempted to solve another problem of the original IR-LEGO, namely, the uncontrollability of the level and duration of the expression of induced genes. In order to generate an IR-LEGO-inducible constitutive expression system, we exploited *in vivo* site-specific recombination systems. The transcription from a promoter is blocked by the presence of an “off cassette,” which consists of a transcriptional terminator flanked by recombinase recognition sites that is inserted between the promoter and the coding region of the desired product. A site-specific recombinase under the control of a heat shock promoter is also introduced as a transgene. Heat shock induces the recombinase, which, in turn, excises the “off cassette” to bring together the promoter and the coding region, leading to expression of the downstream gene (Fig. S3A).

**Figure 3:**
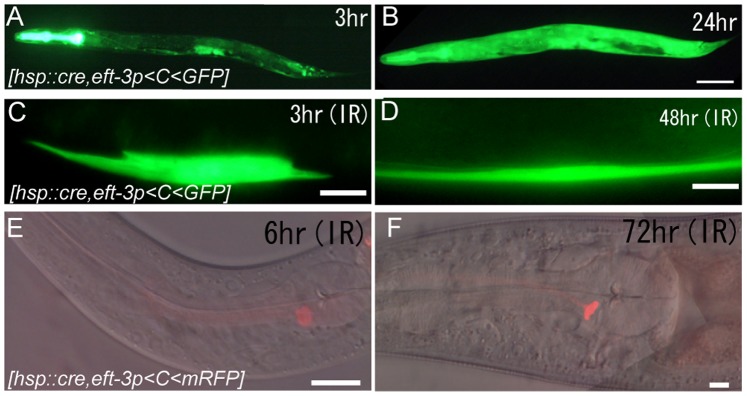
Induction of sustained gene expression using site-specific recombination systems. (A, B) Activation of the recombination reaction with the cre/loxP system following whole-body heat shock treatment. GFP expression mediated by the ubiquitous *eft-3* promoter is detected at 3 h (A) and 24 h (B) following the heat-induced recombination reaction in worms carrying the cre/loxP system *ncIs204[hsp16-2::cre, eft-3p<C<gfp, rol-6(su1006)]*. Scale bar = 50 µm. (C, D) Induced GFP expression in a targeted single body wall muscle cell 3 h and 48 h after IR irradiation in *ncIs204* worms. Scale bars = 10 µm. (E, F) Induced RFP expression in a neuron in the head 6 h and 72 h after IR irradiation in a *ncIs206[hsp16-2::cre, eft-3p<C<mrfp, ajm-1:: gfp]* worm. Scale bars = 10 µm.

First, we compared the recombination in *C. elegans* mediated by two exogenous recombination systems, cre/loxP and FLP/FRT. To visually monitor recombination *in vivo*, we used target constructs consisting of the constitutively active *eft-3*/elongation factor1α gene promoter, “off cassette” of the respective recombination system, and GFP cDNA. Before heat shocking, no GFP signal was detected in worms carrying either the cre/loxP system, *ncIs204[hsp16-2::cre, eft-3p<C<gfp],* or the FLP/FRT system, *ncIs205[hsp16-2::FLP, eft-3p<AB<gfp]* (n>100). Twenty-four hours after heat shocking by placing worm cultures at 37°C for 15 min, GFP was detected in worms carrying either system (Fig. 3A,B, S3B–D), reconfirming that both systems are functional in *C. elegans* [22], [23], [24], [14]. The recombination by the cre/loxP system occurred at a higher frequency in more diverse cell types (Fig. S3D–H, and Text S1).

### Induction of Sustained Gene Expression in Targeted Single Cells Using IR-LEGO

We then irradiated worms carrying the heat-inducible cre/loxP system with IR-LEGO. In *ncIs204[hsp16-2::cre, eft-3p<C<gfp]* worms, approximately 30% of targeted single body wall muscle cells were induced to express GFP (n = 30) and the GFP signal was detectable 2 days after irradiation (Fig. 3C, D). This contrasts with the GFP expression directly driven from a heat shock promoter in *ncIs17* worms, where the GFP signal disappeared almost completely 12 h after irradiation. We also succeeded in inducing a sustained expression of GFP in single DTCs (24%, n = 50) (Fig. S3K, L) and in single neurons (Fig. 3E, F). Sustained GFP expression could also be induced in single neurons using target constructs containing cell-specific promoters instead of *eft-3p* (Fig. S3M–P). Thus, introduction of the cre/loxP system into IR-LEGO has provided a number of options in the expression patterns of the induced gene.

Since the expression of GFP in the above experiments is a result of two processes, that is, induction of heat shock response and excision of the “off cassette” mediated by the recombinase, it occurred less frequently compared with that directly driven by the heat shock promoter. Based on the efficiency of heat shock induction following IR irradiation of targeted DTCs (53%, n = 205) and body wall muscle cells (59%, n = 200) shown in our previous study (Kamei *et al.*, 2009), the estimated recombination rate per successful heat shock induction following IR irradiation of DTCs and body wall muscle cells would be 45% (0.24/0.53) and 51% (0.30/0.59), respectively. Using *ncIs206[hsp16-2::cre; eft-3p<C<mrfp*; *ajm-1::gfp]* worms, we observed sustained induction with IR-LEGO in neurons in the nerve ring in 15% of cases, and estimated the recombination rate to be 25% per successful heat shock induction (see Text S1).

**Figure 4:**
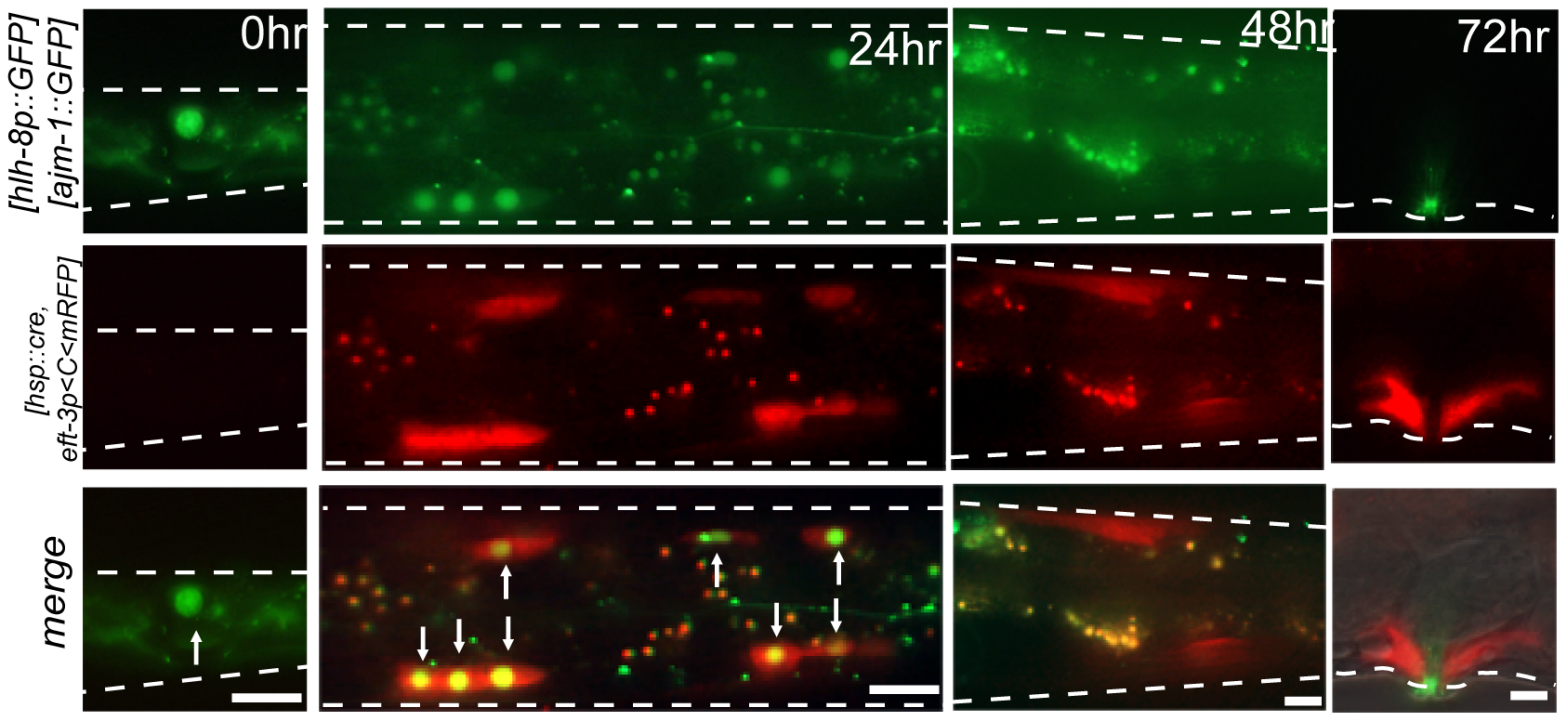
Cell lineage tracing by single cell induction of site-specific recombination. A single M cell, which is visualized with nuclear GFP expressed by the trasngene *hlh-8p::gfp*, was IR-irradiated in *ncIs206[hsp16-2::cre, eft-3p<C<mrfp, ajm-1::gfp]; ayIs6[hlh-8::gfp]* at L1 (0 h). After 24 h, an mRFP signal was detected in body wall muscle cells derived from M at L3. Nuclear GFP signals remain in some M descendants (arrows). Small green dots represent intestinal autofluorescence. An mRFP signal was detected in body wall muscle cells 48 h after irradiation, when the GFP signal disappeared. An mRFP signal was detected in vulva muscles in an adult 72 h after irradiation. The AJM-1::GFP signal indicates the contours of vulva cells. Scale bars = 10 µm.

We then applied the constitutive gene induction system for tracing cell lineages. When an M cell, a larval blast cell producing sex muscles and a subset of body wall muscles [10], was irradiated in *ncIs206[hsp16-2::cre, eft-3p<C<mrfp, ajm-1::gfp]* worms, induction of mRFP expression was detected in 10% of irradiated worms (n = 30). In the induced worms, all M descendants including body wall muscle cells and vulva muscle cells expressed mRFP (Fig. 4), which persisted to adulthood.

To conclude, we have widely extended the applicability of IR-LEGO in *C. elegans* studies. The pulsed irradiation method has not only improved spatial control but has also enabled finer control of heating, enabling us to determine the conditions for efficient gene induction for almost all *C. elegans* somatic cells, including single blastomeres (Table 1). Although we have focused only on the generation of pulses, irradiation can be further tuned by modifying the pulse frequency and duration. Recently, IR-LEGO was successfully used for inducing local gene expression in zebrafish, medaka fish, and *Arabidopsis thaliana* [8], [25]. The innovations reported here would be useful in achieving more efficient, target-specific, and elaborated gene induction in other organisms as well.

**Table 1:** Summary of IR-LEGO irradiation conditions for gene induction in various cell types.

	continuous/pulse	power	duration
seam cells (epidermal cells)	continuous	11 mW	1 s
	8.3-msec pulse at 6 Hz	20 mW	4 s
DTCs (distal tip cells)	continuous	11 mW	0.25 s×4
body wall muscles	continuous	11 mW	1 s
M-cells	8.3-msec pulse at 6 Hz	12 mW	4 s
Neurons			
AIY (nerve ring neuron)	8.3-msec pulse at 6 Hz	12 mW	4 s
	0.83-msec pulse at 60 Hz	16 mW	4 s
ALM (sensory neuron on the lateral body)	continuous	12 mW	0.5 s
	8.3-msec pulse at 6 Hz	16 mW	4 s
motor neurons in VNC	continuous	13 mW	0.5 s
	0.83-msec pulse at 60 Hz	16 mW	4 s
Embryos			
2-cell	8.3-msec pulse at 6 Hz	23 mW	4 s×2
4-cell	8.3-msec pulse at 6 Hz	18 mW	4 s×2
8-cell	8.3-msec pulse at 6 Hz	18 mW	4 s
16-cell	8.3-msec pulse at 6 Hz	17 mW	4 s

## Materials and Methods

### Strains

*C. elegans* were cultured according to standard methods [26]. The strains used in this study are as follows:

N2 (wild-type),

*ncIs17[hsp16-2::gfp],*

*ncIs201[hsp16-2::gfp, ttx-3p::mrfp, rol-6(su1006)],*

*ncIs204[hsp16-2::cre, eft-3p<C<gfp, rol-6(su1006)],*

*ncIs205[hsp16-2::FLP, eft-3p<AB<gfp, rol-6(su1006)],*

*ncIs206[hsp16-2::cre, eft-3p<C<mrfp, ajm-1:: gfp],*

*ncEx2001[hsp16-2::cre, mec-7p<D<gfp, rol-6(su1006)],*

*ncEx2002[hsp16-2::cre, H20<D<gfp, rol-6(su1006)],*

*ncEx2003[hsp16-2::FLP, mec-7p<AB<gfp, rol-6(su1006), mec-7p::mrfp],*

*ncEx2004[hsp16-2::FLP, H20<AB<gfp, rol-6(su1006)],*

*ncIs203[H20::mrfp],*

*ayIs6[hlh-8::gfp],*

*him-8 (e1489): LGIV,*

Transgenic lines were generated by microinjection [27], using *rol-6(su1006)* or *myo-2p::mrfp* as the transformation marker (see Text S1). Integrated strains were generated by γ-ray-irradiation [28].

### Constructs

pPDEF1α (a gift from Yasumi Ohshima and Makoto Koga) contains a *Xba*I-*Bam*I 2.7 kb fragment upstream to the *eft-3* gene cloned into pPD49.26 [29], and is used as a vector for driving constitutive expression. *FRT* “off cassette” *AB* consists of *FRT::AUG:: β-gal::FRT* in pC>AB> [30]. *loxP “*off cassettes” *C* and *D* each consist of *loxP::SCS::loxP* and *loxP::mrfp::SCS::loxP,* and were constructed from *PHR68*, a derivative of *pHR56* containing a *loxP* site-flanked PGK-TKneoA+ cassette [31]. *C* and *D* both contain the *Drosophila melanogaster* special chromatin structure (SCS) fragment, which is thought to function as a transcription insulator. Polymerase chain reaction (PCR)-amplified FLP cDNA and cre cDNA was inserted into the *KpnI* site of pPD49.78 (Fire Vector kit) to generate *hsp16-2::FLP* and *hsp16-2::cre,* respectively. (See Text S1 for details of plasmid construction).

### Microscope and Related Equipment

The IR-LEGO setup was described previously. In brief, an IR laser (Model FRL-DC 1,480 nm, maximum power of 3 W, IRE-Polus, Russia) was connected to an inverted microscope (IX-70 with a mono-coated UApo/340 100× oil [NA = 1.3] objective, Olympus, Japan). For pulsed irradiation, an optical chopper (microprocessor-controlled rotating blade with an opening of 18 degrees; MC1000A, Thorlabs, USA) was installed on the laser beam path.

### IR Laser Irradiation of Worms

Individual worms were mounted on a 6% agar pad of approximately 0.2 mm thickness with levamisole (0.1 mM to 1 mM, Nacalai, Japan) to immobilize them, and were then covered with a coverslip (microcover glass, 0.12–0.17 mm in thickness, Matsunami, Japan). (See Table 1 and Text S1 for a summary of conditions for irradiation of different cell types.) After irradiation, worms were returned to the original plates and left at 20°C. For IR irradiation experiments, images were captured by a cooled charge coupled device (CCD) camera (MC681-SPD-ROBO, Texas Instruments, USA). Worms were observed with a Zeiss Axioplan microscope (Zeiss, Germany). Confocal images of embryos were captured with an Olympus Fluoview 1000 laser scanning microscope (Olympus, Japan).

## Supporting Information

Figure S1 **Continuous and pulsed irradiation of seam cells (related to** **Fig. 1** **).**
(TIF)

Figure S2 **Gene induction in targeted single cells of embryos (related to** **Fig. 2** **).**
(TIF)

Figure S3 **Induction of sustained gene expression in targeted single cells using IR-LEGO (related to** **Fig. 3** **).**
(TIF)

Table S1 **Gene induction in comma-stage embryos.**
(DOC)

Text S1 **Materials and Methods.** Constructs, Generation of transgenic animals, IR laser irradiation of worms, Site-specific recombination in *C. elegans.*
(DOC)

Text S2 **Legends for Supporting Figures and a Supporting Table.**
(DOC)
