# Supplementary figures and images for: Pulsed Irradiation Improves Target Selectivity of Infrared Laser-Evoked Gene Operator for Single-Cell Gene Induction in the Nematode C. elegans
Source: PLoS One. 2014 Jan 20;9(1):e85783. doi: 10.1371/journal.pone.0085783 (PMC3896399; doi:10.1371/journal.pone.0085783)

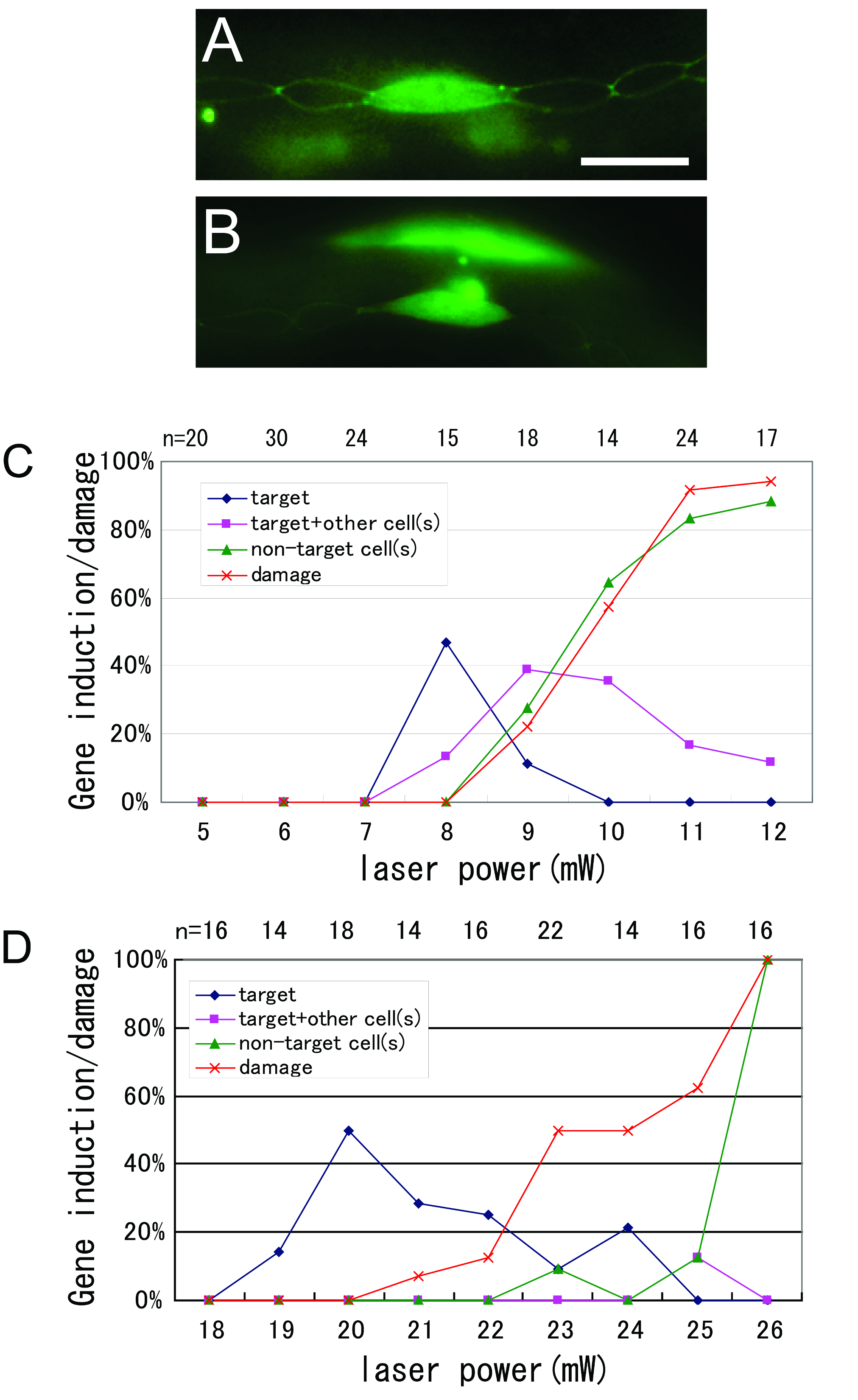

Supplement: Figure S1 — Continuous and pulsed irradiation of seam cells (related to Fig. 1 ). (TIF) [file pone.0085783.s001.tif]

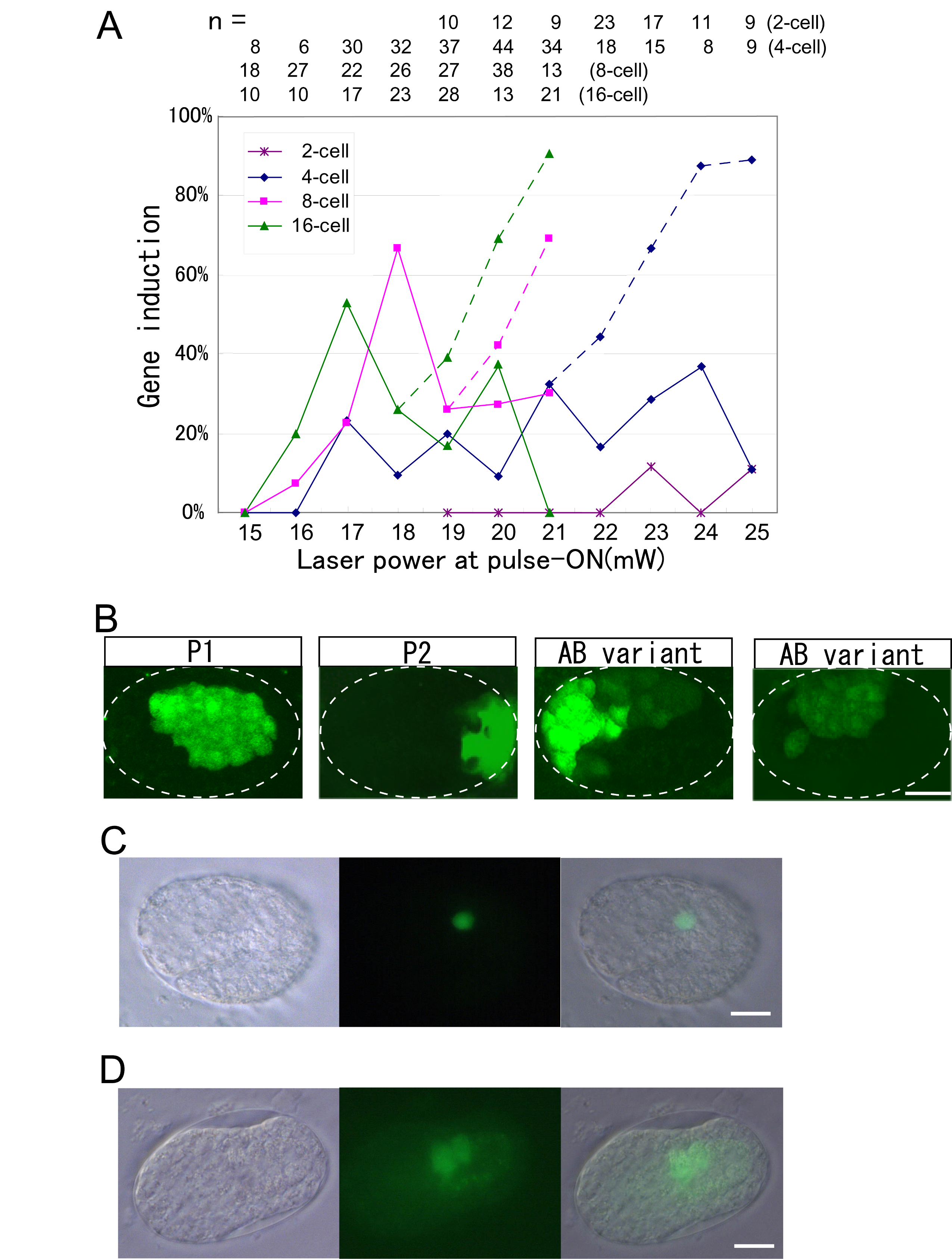

Supplement: Figure S2 — Gene induction in targeted single cells of embryos (related to Fig. 2 ). (TIF) [file pone.0085783.s002.tif]

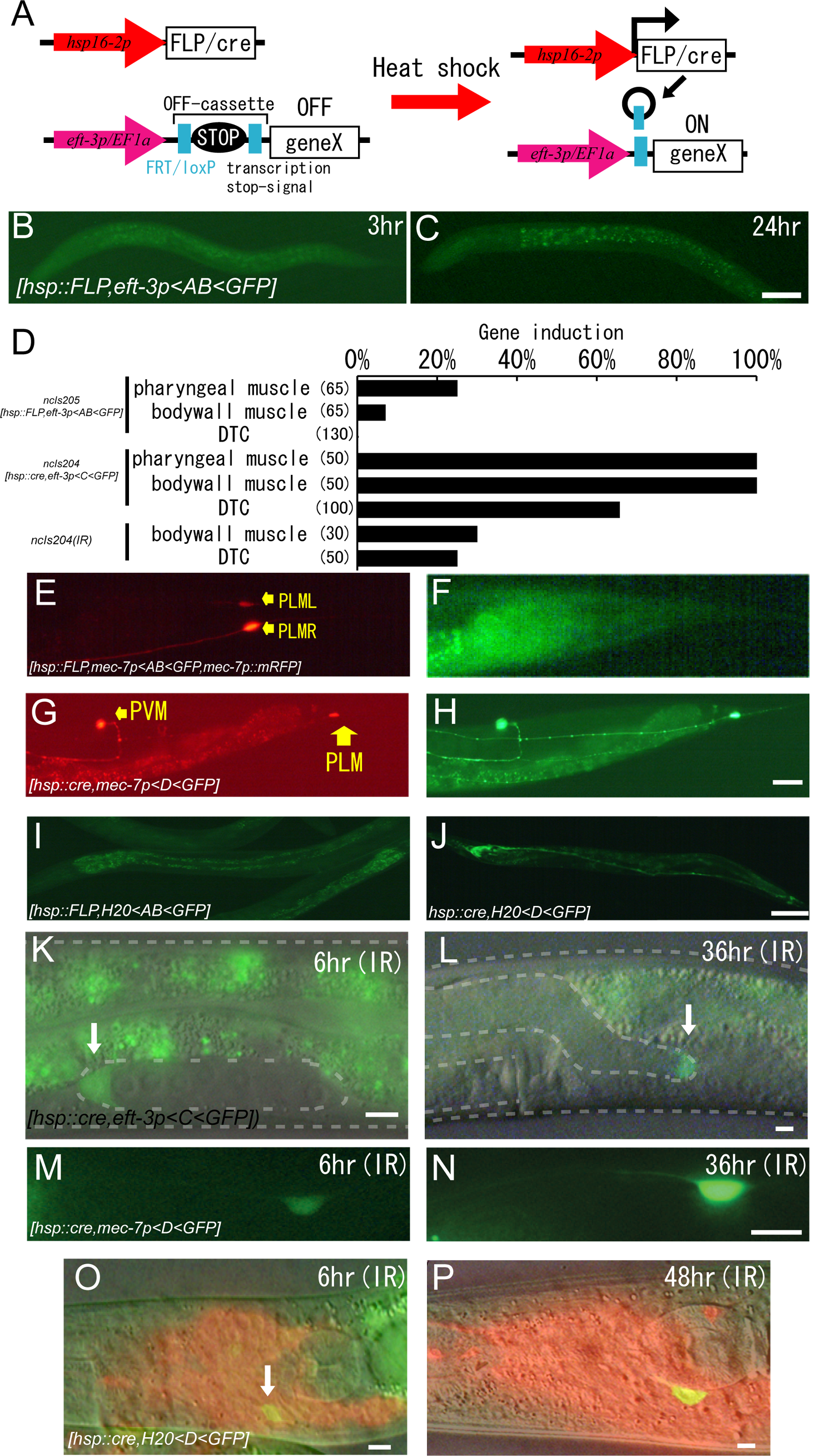

Supplement: Figure S3 — Induction of sustained gene expression in targeted single cells using IR-LEGO (related to Fig. 3 ). (TIF) [file pone.0085783.s003.tif]
