# Supplementary material for: Pulsed Irradiation Improves Target Selectivity of Infrared Laser-Evoked Gene Operator for Single-Cell Gene Induction in the Nematode C. elegans
Source: PLoS One. 2014 Jan 20;9(1):e85783. doi: 10.1371/journal.pone.0085783 (PMC3896399; doi:10.1371/journal.pone.0085783)
Supplement: Table S1 — Gene induction in comma-stage embryos. (DOC) [file pone.0085783.s004.doc]

**Table S1**

Induction of GFP in comma-stage embryos

| Laser power  at pulse-ON | GFP induction in | | arrested | total |
| --- | --- | --- | --- | --- |
|  | single cell | multiple cells | embryos | embryos |
| 12 mW | 0 | 0 | 0 | 17 |
| 13 mW | 3 | 0 | 0 | 19 |
| 14 mW | 1 | 8 | 0 | 13 |
| 15 mW | 0 | 4 | 0 | 19 |
| 16 mW | 0 | 13 | 6 | 18 |
| 17 mW | 0 | 13 | 8 | 17 |
| 18 mW | 0 | 9 | 13 | 13 |
