# Supplementary material for: Pulsed Irradiation Improves Target Selectivity of Infrared Laser-Evoked Gene Operator for Single-Cell Gene Induction in the Nematode C. elegans
Source: PLoS One. 2014 Jan 20;9(1):e85783. doi: 10.1371/journal.pone.0085783 (PMC3896399; doi:10.1371/journal.pone.0085783)
Supplement: Text S1 — Materials and Methods. Constructs, Generation of transgenic animals, IR laser irradiation of worms, Site-specific recombination in C. elegans. (DOC) [file pone.0085783.s005.doc]

**Text S1**

# Materials and Methods

### *Constructs*

For the construction of the *eft-3p::gfp*, the *Kpn*I-*Apa*I fragment of pPD95.75 (a gift from Andrew Fire), including egfp cDNA and unc-54 3’UTR, was ligated into pPDEF1α digested with *Kpn*I and *Apa*I. To create *mec-7p::gfp,* a *Kpn*I and *Apa*I fragment of pPD95.75 (a gift from Andrew Fire), including egfp cDNA and *unc-54* 3’UTR, was ligated between the *Kpn*I and *Apa*I site of pPD53.102 (a gift from Andrew Fire) carrying the *mec-7* promoter. To create *H20::mrfp*, the *Kpn*I-*Apa*I fragment of pHK205 (a gift from Hiroshi Kagoshima) was inserted into H20 digested with *Kpn*I and *Apa*I (Shioi et al., 2001).

*AB*, *C* and *D* represent the *FRT* “off cassette”, *loxP* “off cassette”, and *loxP* “off cassette” containing promoter-less *mrfp,* respectively.

The *FRT* “off cassette” *AB* consists of *FRT::AUG::β-gal::FRT* in pC>AB> (Wimmer et al., 1997). A transcriptional stop signal of hsp70 is also present downstream of β-GAL in *AB*. To generate the constructs *eft-3p<AB<gfp* and *mec-7p<AB<gfp,* a 4.0kb *Spe*I-*Nhe*I fragment of pC>AB> was cloned into the *Nhe*I sites of *eft-3p::gfp* and *mec-7p::gfp*.

The *loxP “*off cassettes” *C* and *D* each consist of *loxP::SCS::loxP* and *loxP::mrfp::SCS::loxP,* and were constructed from *PHR68*, a derivative of *pHR56* containing a *loxP* site-flanked *PGK-TKneoA+* cassette (Metzger et al., 1995). The *Drosophila melanogaster SCS* (Special Chromatin Structure) fragment was amplified with the PCR primers 5’-ttACTAGTagctgcgctgcgaacttctc-3’ and 5’-gaCTGCAGctgtattcctcagttatcg-3’ from *Drosophila* genomic DNA (a gift from Katsumasa Yagi). To generate the *loxP “*off cassette” *C* and the *loxP “*off cassette” *D*, we first subcloned the *BamH*I-*Sma*I fragment of PHR68 including *loxP::PGK-TKneoA+::loxP* into the *BamH*I-*EcoR*V site of pBlueskriptKS (Stratagene, USA). Then we removed the *EcoR*V-*Sca*I fragment from the *PGK 3’UTR*. Then, the *SCS* fragment was inserted between the *Spe*I and *Pst*I sites. The *Xba*I fragment was excised and cloned into the *Nhe*I site of *eft-3p*::*gfp* and *mec-7p:: gfp* to create *eft-3p<C< gfp* and *mec-7p<C< gfp*, respectively. In the *loxP off* cassette *D,* the *Sma*I-*Spe*I fragment of *pHK210* (a gift from Hiroshi Kagoshima) containing the mRFP cDNA was inserted into the blunt-ended *EcoR*I and *Spe*I sites of the *loxP off* cassette *C*.

To create *H20<AB<gfp*, and *H20<D<gfp,* the *Pst*I fragment of H20 was first subcloned into pPD49.26. Then, the *Kpn*I-*Apa*I fragment of pPD95.75 (a gift from Andrew Fire) was subcloned into the resulting plasmid, which was then digested with *Nhe*I site and ligated with the *Xba*I-site flanked *FRT “*off cassette” *AB* and *loxP “*off cassette” *D*.

Next, *cre* was derived from pCrebpA. To construct *hsp16-2::cre*, a PCR fragment amplified from pCrebpA with the primers 5’-ATggtaccgctagcaaaaATGGCCAATTTACTGACCGT-3’ and 5’-atGGTACCctaatcgccatcttccagcaggcgcaccat-3’ was digested with *Kpn*I and ligated into the *Kpn*I site of pPD49.78 carrying a heat shock promoter, *hsp16-2,* and *unc-53* 3’UTR (a gift from Andrew Fire).

*FLP* was derived from pCP20 (Cherepanov and Wackernagel, 1995). For the construction of *hsp16-2::FLP*, the FLP cDNA was amplified from pCP20 by PCR using the primers 5’-Atggtaccaaaatgccacaatttggtatattatgt-3’ and 5’-taggtaccttatatgcgtctatttatgtA-3’, and was cloned into the *Kpn*I site of pPR49.78.

The plasmid carrying *ttx-3p::gfp,* an AIY marker, was a gift from Ikue Mori.

# *Generation of transgenic animals*

Weight ratios of plasmids in the injection mixtures were as follows:

*hsp16-2::cre* : *mec-7p<D<gfp* : *pRF4* =1:1:1 for *ncEx2001[hsp16-2::cre, mec-7p<D<gfp, rol-6(su1006)]*

*hsp16-2::cre* : *H20<D<gfp,*: *pRF4* =1:1:1 for *ncEx2002[hsp16-2::cre, H20<D<gfp, rol-6(su1006)]*

*hsp16-2::FLP* : *mec-7p<AB<gfp* : *pRF4* : *mec-7p::mrfp* =1:1:2:1 for *ncEx2003[hsp16-2::FLP, mec-7p<AB<gfp, rol-6(su1006), mec-7p::mrfp]*

*hsp16-2::FLP* : *H20<AB<gfp* : *pRF4* =1:1:2 for *ncEx2004[hsp16-2::FLP, H20<AB<gfp, rol-6(su1006)]*

*hsp16-2::gfp* : *ttx-3p::mrfp* : *pRF4* =1:1:1 for *ncIs201[hsp16-2::gfp, ttx-3p::mrfp, pRF4]*

*hsp16-2::cre* : *eft-3p<C<gfp* : *pRF4[rol-6(su1006)*=3:1:3 for *ncIs204[hsp16-2::cre, eft-3p<C<gfp, rol-6(su1006)]*

*hsp16-2::FLP* : *eft-3p<AB<gfp* : *pRF4[rol-6(su1006)*=4:1:4 for *ncIs205[hsp16-2::FLP, eft-3p<AB<gfp, rol-6(su1006)]*

*hsp16-2::cre* : *eft-3p<C<mrfp* : *pJS191[ajm-1::gfp]* =30:30:1 for *ncIs206[hsp16-2::cre* : *eft-3p<C<mrfp* : *ajm-1:: gfp]*

# *IR-laser irradiation of worms*

For irradiation of different cell types, optimum conditions for gene induction varied, and these were determined empirically (Table 1): In DTCs, 4 consecutive applications of continuous IR-laser irradiation at 11 mW each for 0.25 s was sufficient for gene induction. For body wall muscle cells, continuous irradiation at 11 mW for 1 s was used. For neurons in the nerve ring, pulsed irradiation (8.3-msec pulse width, 6 Hz) at 12 mW for 4 s, or pulsed irradiation (833-μsec pulse width, 60 Hz) at 16.4 mW for 4s was used. For M cells, pulsed irradiation (8.3-msec pulse width, 6 Hz) at 12 mW for 4 s was used. For 8-, 16-cell and comma-stage embryos, we used pulsed irradiation (8.3-msec pulse width, 6 Hz) for 4 s at 18 mW, 17 mW, and 14 mW, respectively. We found that two applications of pulsed irradiation for 4 s each with a 1-s interval between applications induced gene expression efficiently in 2- and 4-cell embryos at power levels of 23 mW and 18 mW, respectively. Application of irradiation twice with a brief interval between applications generally increased the efficiency of gene induction in most cells.

For immobilizing worms during irradiation, levamisole solution at a concentration between 0.1 and 5 mM was used. A very high concentration of levamisole, e.g., 10 mM, caused developmental arrest in almost all cases. Another measure for suppressing the motile response of worms during irradiation involves mounting worms on a pad containing a high concentration of agar. We usually use a 6% agar pad. The pulsed irradiation method, in addition to offering better spatial control of heating compared with continuous irradiation, offers another advantage: We noticed that worms sometimes moved slightly during IR-irradiation, which probably shifted the focus of irradiation to non-targeted cells. Compared to continuous irradiation, pulsed irradiation elicited motile response in worms less frequently, allowing us to target cells more consistently and accurately.

***Site-specific recombination in* C. elegans**

Two exogenous site-specific recombination systems, cre/loxP and FLP/FRT, were used in this study. The respective recombinases under the control of a heat shock promoter were induced through the heat shock response, and subsequent recombination events were detected by expression of GFP or mRFP from marker plasmids containing the target sequences of the recombinase. For heat shocking of a whole animal, worm culture plates were placed in an air-incubator at 37 °C for 15 min, and then they were returned to an incubator at 20°C. This treatment is sufficient for inducing GFP expression in almost all somatic cells in *ncIs17[hsp16-2::gfp]* animals.

In some transgenic strains carrying the *loxP* “off cassette” with *hsp16-2::cre* or the *FRT* “off cassette” with *hsp16-2::FLP* , GFP expression was detected in a few cells even without heat shocking. This “leaky” expression of GFP was occasionally observed in some *ncEx* strains carrying the transgene as an extrachromosomal array, but not in most of the *ncIs* strains carrying the chromosomally integrated transgene. Thus, for the induction experiments, we generated multiple transgenic lines and tried to select the ones that do not exhibit leaky GFP expression without heat shocking. Also, we found it important to keep worms healthy, as starvation often triggers leaky GFP expression. Before applying a heat shock treatment to a whole animal, we confirmed that no GFP/mRFP signal was detected in *ncIs* strains, which are used in this study, carrying either the cre/loxP system or the FLP/FRT system as a chromosomally integrated transgene.

Twenty-four hours after heat shocking, we scored the recombination events in *ncIs204[hsp16-2::cre, eft-3p<C<gfp, rol-6(su1006)]* worms carrying the cre/loxP system and in *ncIs205[hsp16-2::FLP, eft-3p<AB<gfp, rol-6(su1006)]* worms carrying the FLP/FRT system. While expression of GFP, an indicator of the recombination events, was detected in pharyngeal muscles and body wall muscles in both strains, GFP was expressed much more frequently in *ncIs204* worms. In DTCs, recombination events were detected in 65% of *ncIs204* worms but in none of *ncIs205* worms (Fig. S3D)*.* We also examined the recombination events in touch receptor neurons using, instead of *eft-3p*, the promoter of the *mec-7* gene, which encodes a beta-tubulin required for touch sensitivity along the body wall and is expressed in all six touch neurons (Fig. S3E-H). Six hours after heat shocking of a whole animal, about 95% of touch neurons in *ncEx2001[hsp16-2::cre, mec-7p<D<gfp, rol-6(su1006)]* worms carrying the cre/loxP system were induced to express GFP (n = 323), while no GFP signal was detected in *ncEx2003[hsp16-2::FLP, mec-7p<AB<gfp, rol-6(su1006), mec-7p::mrfp]* worms carrying the FLP/FRT system (n = 320).

We found that the recombination by the cre/loxP system occurred at a higher frequency in more diverse cell types compared with the FLP/FRT system. This agrees with previous studies that compared the two systems *in vitro* (Buchholz et al., 1996; Ringrose et al., 1998), in cultured cells (Nakano et al., 2001), in *Xenopus* embryos (Werdien et al., 2001) and in zebrafish (Boniface et al., 2009). Whereas a previous study showed that the FLP/FRT system is useful for heat-induced recombination in neurons of *C. elegans* (Davis et al., 2008, Voutev and Hubbard, 2008), we failed to detect FLP/FRT-mediated recombination events in neurons. The disagreement may be due to differences in the construction of the plasmids containing the FLP/FRT system, the promoters used to drive a marker expression, and/or our milder heat shock protocol.

Under identical irradiation conditions, gene induction mediated by the cre/loxP recombination system occurred less frequently compared with that directly driven by the heat shock promoter: Pulsed-irradiation of an unidentified single neuron in the nerve ring in *ncIs17* worms with 8.3-msec pulse width at 6 Hz and 11 mW for 4 s led to induction of marker GFP in about 60% of worms (n = 34). On the other hand, irradiation of an unidentified single neuron in the nerve ring of *ncIs206[hsp16-2::cre; eft-3p<C<mrfp*; *ajm-1::gfp]* worms under the same conditions led to expression of marker RFP in about 15% of worms (n = 52), indicating that the estimated recombination rate per successful heat shock induction is about 25% (0.15/0.60). The relatively low value of the estimated recombination rate may reflect the fact that the intensity of the heat shock response induced by IR-LEGO shows a wider variation compared to that induced by whole-body heat shock treatment.

In *ncEx2002[hsp16-2::cre, H20<D<gfp, rol-6(su1006)]*, GFP was sometimes detected in a small number of neurons before heat shocking, suggesting the somewhat leaky expression of cre recombinase in this strain*.* When we performed IR-irradiation of *ncEx2002* worms, individuals were used after we had checked that no leaked GFP expression was detected in the head.

We tried lineage tracing of early embryonic cells in *ncIs204[hsp16-2::cre, eft-3p<C<gfp]*, but all the embryos that were successfully induced to express GFP showed arrested development before hatching. Either activation of the cre/loxP system, overexpression of cre recombinase itself, and/or GFP seems to affect the embryonic development in *ncIs204*.

# References for Supporting Information

Buchholz, F., Ringrose, L., Angrand, P. O., Rossi, F., and Stewart, A. F. (1996). Different thermostabilities of FLP and Cre recombinases: implications for applied site-specific recombination. Nucleic Acids Res 24: 4256-4262.

Boniface, E. J., Lu, J., Victoroff, T., Zhu, M., and Chen, W. (2009). FlEx-based transgenic reporter lines for visualization of Cre and Flp activity in live zebrafish. Genesis 47: 484-491.

Cherepanov, P. P., and Wackernagel, W. (1995). Gene disruption in Escherichia coli: TcR and KmR cassettes with the option of Flp-catalyzed excision of the antibiotic-resistance determinant. Gene 158: 9-14.

Davis, M. W., Morton, J. J., Carroll, D., and Jorgensen, E. M. (2008). Gene activation using FLP recombinase in C. elegans. PLoS Genet 4: e1000028.

Kamei, Y., Suzuki, M., Watanabe, K., Fujimori, K., Kawasaki, T., Deguchi, T., Yoneda, Y., Todo, T., Takagi, S., Funatsu, T., and Yuba, S. (2009). Infrared laser-mediated gene induction in targeted single cells in vivo. Nat Methods 6: 79-81.

Metzger, D., Clifford, J., Chiba, H., and Chambon, P. (1995). Conditional site-specific recombination in mammalian cells using a ligand-dependent chimeric Cre recombinase. Proc Natl Acad Sci U S A 92: 6991-6995.

Nakano, M., Odaka, K., Ishimura, M., Kondo, S., Tachikawa, N., Chiba, J., Kanegae, Y., and Saito, I. (2001). Efficient gene activation in cultured mammalian cells mediated by FLP recombinase-expressing recombinant adenovirus. Nucleic Acids Res 29: E40.

Ringrose, L., Lounnas, V., Ehrlich, L., Buchholz, F., Wade, R., and Stewart, A. F. (1998). Comparative kinetic analysis of FLP and cre recombinases: mathematical models for DNA binding and recombination. J Mol Biol 284: 363-384.

Shioi, G., Shoji, M., Nakamura, M., Ishihara, T., Katsura, I., Fujisawa, H., and Takagi, S. (2001). Mutations affecting nerve attachment of Caenorhabditis elegans. Genetics 157: 1611-1622.

Voutev, R., and Hubbard, E. J. (2008). A "FLP-Out" system for controlled gene expression in Caenorhabditis elegans. Genetics 180: 103-119.

Werdien, D., Peiler, G., and Ryffel, G. U. (2001). FLP and Cre recombinase function in Xenopus embryos. Nucleic Acids Res 29: E53-53.

Wimmer, E. A., Cohen, S. M., Jackle, H., and Desplan, C. (1997). buttonhead does not contribute to a combinatorial code proposed for Drosophila head development. Development 124: 1509-1517.
