# Supplementary material for: Pulsed Irradiation Improves Target Selectivity of Infrared Laser-Evoked Gene Operator for Single-Cell Gene Induction in the Nematode C. elegans
Source: PLoS One. 2014 Jan 20;9(1):e85783. doi: 10.1371/journal.pone.0085783 (PMC3896399; doi:10.1371/journal.pone.0085783)
Supplement: Text S2 — Legends for Supporting Figures and a Supporting Table. (DOC) [file pone.0085783.s006.doc]

**Text S2**

**Legends for Supporting Figures and a Supporting Table**

**Fig. S1.** **Continuous and pulsed irradiation of seam cells**

Seam cells in *ncIs17[hsp16-2::gfp]* worms at the fourth larval stage (L4) were irradiated with IR-laser for 4 s at various power levels either continuously or in pulses.

(A,B) Continuous irradiation sometimes led to GFP induction exclusively in targeted seam cells (A), but it often caused GFP expression in non-targeted cells such as body wall muscle cells (top) in addition to the targeted seam cells (bottom) (B). Scale bar = 10 μm.

(C) Gene induction index and cell damage index against incident laser power for continuous irradiation for 4 s of single L4 seam cells in *ncIs17*. Blue diamonds indicate GFP expression only in the target cell; magenta squares indicate gene expression index where both the target single-cell and non-target cell(s) were induced simultaneously. Green triangles indicate gene expression only in non-target cell(s). Gene induction was measured 3 h after laser-irradiation. Red crosses represent the rate of damaged cells, recognized by aberrant patterns of the AJM-1::GFP expression 3 h after irradiation. Numbers of trials are shown at the upper margin.

Continuous irradiation with incident laser power at 8 mW led to GFP induction exclusively in targeted seam cells in about 50% of worms, but in 10% of irradiated worms, GFP expression was also induced in non-targeted cells in addition to the targets. At 9 mW for 4 s, 50% of irradiated seam cells expressed GFP, but non-targeted cells such as body wall muscle cells also expressed GFP in most cases. At powers higher than 10 mW, irradiated cells were frequently damaged after irradiation and non-targeted cells were induced to express GFP. These results confirmed our previous observation with continuous irradiation at high power, namely, that heat not only harms the target but is also transferred to surrounding areas to induce gene expression in cells in the vicinity of the targets (Kamei et al., 2009).

(D) Gene induction index and cell damage index against incident laser power for irradiation with 8.3 ms laser pulses for 4 s at 6 Hz of single L4 seam cells in *ncIs17*. With pulsed irradiation for 4 s at 20mW during the pulse-on period, irradiated seam cells were exclusively induced to express GFP in 50% of worms (blue diamonds). At higher power, irradiated cells sometimes appeared to be damaged, but GFP expression in non-targeted cells was observed only rarely (magenta squares).

## **Fig. S2.** **Gene induction in targeted single cells of embryos**

(A) Gene induction index and cell damage index for embryonic cells against incident laser power for irradiation of single cells in *ncIs17* with 8.3 ms laser pulses for 4 s at 6 Hz. Results of irradiation of 16-cell (green triangles), 8-cell (magenta squares), 4-cell (blue circles) and 2-cell (red crosses) stage embryos are shown. Note that in this experiment, a single cell in embryos was irradiated randomly irrespective of its identity. Solid lines indicate the cases where cells expressing GFP developed normally, and dashed lines indicate the total cases where GFP expression was observed, regardless of whether embryos suffered arrested development or continued to develop normally.

(B) GFP expression in variable patterns was observed with IR-irradiation of certain blastomeres. GFP expression was detected in EMS descendants and C descendants when the germ-line blastomere P1 and P2 were IR-irradiated just before their cell division (far-left and middle-left panels). Variable expression patterns were also observed with IR-irradiation of AB (far-right and middle-right panels). GFP was expressed in combinations of descendants of ABal, ABar, ABp or ABp. GFP expression in ABal descendants was often the strongest. These results imply that activated heat shock factors and/or the *gfp* messages are sometimes distributed asymmetrically to daughters and granddaughters of AB, when heat shock is applied just prior to the cleavage of AB.

(C) Induced GFP expression following IR-irradiation of a comma-stage embryo. A cell in the comma-stage embryo was randomly targeted. A lateral view showing a single cell expressing GFP 3 h after irradiation. Left: the bright field image; middle: the fluorescent image; right: a composite of the left and middle images.

(D) Induced GFP expression following IR-irradiation of a comma-stage embryo. A ventral view showing two intestine cells expressing GFP 3 h after irradiation. Left: the bright field image; middle: the fluorescent image; right: a composite of the left and middle images. Scale bars = 10 μm.

**Fig. S3. Induction of sustained gene expression in targeted single cells using IR-LEGO**

(A) Schematics of constructs for site-specific recombination in *C. elegans* and strategy for inducing continuous gene expression. (Left top) Expression of site-specific recombinase (FLP or cre) is under the control of a heat shock promoter. (Left bottom) In the target construct, the “off-cassette” flanked by recombinase target sequences (FRT or loxP) is inserted between a constitutive promoter and gene X, whose expression is blocked by the “off-cassette.” (Bottom) Heat-induced recombinase excises the “off-cassette” and activates the expression of gene X.

(B, C) Activation of recombination reaction with the FLP/FRT system.

GFP expression mediated by the ubiquitous *eft-3* promoter at 3 h (B) and 24 h (C) after the heat-induced recombination reaction in worms carrying the FLP/FRT system *ncIs205[hsp16-2::FLP, eft-3p<AB<gfp, rol-6(su1006)]*. GFP expression triggered by recombination mediated by the FLP/FRT system is very weak and hardly recognizable at low magnification, whereas that mediated by the cre/loxP system occurs intensely in diverse cell types (see Fig. 3A, B). (B, C) Scale bar = 50 μm.

(D) Gene induction index for pharyngeal muscle cells, body wall muscle cells and distal tip cells (DTC) in *ncIs205[hsp16-2::FLP, eft-3p<AB<gfp, rol-6(su1006)]* (top) and *ncIs204[hsp16-2::cre, eft-3p<C<gfp, rol-6(su1006)]* (middle) by conventional heat shock treatment to whole animals by heating worm culture plates to 37 °C for 20 min. We scored worms that expressed GFP, an indicator of recombination events, in more than one cell in the relevant tissues. Numbers of total worms examined are shown in parentheses. Twenty-four hours after heat shocking of *ncIs205* worms carrying the FLP/FRT system (top), the GFP signal was detected mainly in pharyngeal muscles (25%, n = 65). Body wall muscle cells expressed GFP at a low frequency (6%, n = 65). On the other hand, in *ncIs204* worms carrying the cre/loxP system 24 h after heat shocking (middle), GFP induction was observed frequently in a variety of tissues, including pharyngeal muscles, body wall muscles, and DTCs (100%, n = 50; 100%, n = 50; and 65%, n = 100, respectively), and the signal was detected even 2 days after heat shocking. In worms with the cre/loxP system, GFP expression was observed frequently in pharyngeal muscles, body wall muscles and DTCs (97%, 34%, and 30% respectively) as early as 3 h after heat shocking, when no GFP signal was detected in worms with the FLP/FRT system. The gene induction index of IR-irradiation of single body wall muscle cells and single DTCs in *ncIs204* is shown at the bottom.

(E-H) Induced GFP expression under the control of the touch cell-specific *mec-7* promoter in *ncEx2003[hsp16-2::FLP, mec-7p<AB<gfp, rol-6(su1006), mec-7p::mrfp]* (E, F) and an *ncEx2001[hsp16-2::cre, mec-7p<D<gfp, rol-6(su1006)]* (G,H) worms after heat shock treatment of the whole animal. (E, G) Touch neurons were marked with *mec-7p::mrfp*. In *ncEx2001,* mRFP was expressed from the *D* cassette. Before heat shocking, GFP expression was not observed. (F) In worms carrying the FLP/FRT system, the GFP signal was seldom detected after heat shocking. (H) GFP expression was induced in PVM and PLM touch neurons in *ncEx2003* worms carrying the cre/loxP system. The intestine granules showed background green autofluorescence. Scale bar = 10 μm.

(I, J) Induction of GFP expression under the control of a pan-neuronal H20 promoter following the heat shock treatment of the whole animal. (J) In *ncEx2002[hsp16-2::cre, H20<D<gfp, rol-6(su1006)]* worms 3 h after heat shocking, GFP was expressed in the entire nervous system. (I) GFP expression was not observed in an *ncEx2004[hsp16-2::FLP, H20<AB<gfp, rol-6(su1006)]* worm after heat shocking. The intestine granules show green background autofluorescence. Scale bar = 50 μm.

(K, L) Induced GFP expression in an IR-irradiated DTC (arrow) of the gonad arm in an *ncIs204[hsp16-2::cre, eft-3p<C<gfp, rol-6(su1006)]* worm 6 h (K) and 36 h (L) after irradiation with IR-LEGO at 11 mW for 1 s. A Nomarski and a GFP image were merged. The intestine granules show green background autofluorescence. Scale bar = 10 μm.

(M, N) GFP expression induced by IR-irradiation in a targeted PLM neuron under the control of the touch neuron specific *mec-7* promoter in an *ncEx2001[hsp16-2::cre, mec-7p<D<gfp, rol-6(su1006)]* worm 6 h (M) and 36 h (N) after irradiation with IR-LEGO at 12 mW for 0.5 s. Scale bar = 5 μm.

(O, P) Induced GFP expression in a single nerve ring neuron (arrow) under the control of the pan-neuronal *H20* promoter in an *ncEx2002[hsp16-2::cre, H20<D<gfp, rol-6(su1006)]* worm 6 h (O) and 48 h (P) after irradiation with IR-LEGO with 8.3 ms laser pulses for 4 s at 6 Hz at the laser power level of 11 mW during the pulse-on period. Many neurons in the head exhibited mRFP signal expressed from the lox-P “off cassette“ *D*. A Nomarski, a GFP and an mRFP image were merged. Scale bar = 5 μm.

**Table S1. Induction of GFP in comma-stage embryos**

Single cells in *ncIs17[hsp16-2::gfp]* embryos at the comma stage were irradiated at random for 4 s with 8.3-msec laser pulses at 6 Hz at various power levels.
